# Mechanical versus enzymatic isolation of stromal vascular fraction cells from adipose tissue

**DOI:** 10.1186/s40064-015-1509-2

**Published:** 2015-11-23

**Authors:** Joel A. Aronowitz, Ryan A. Lockhart, Cloe S. Hakakian

**Affiliations:** Cedars-Sinai Medical Center, Los Angeles, USA; University Stem Cell Center, 8635 W 3rd St. Suite 1090W, Los Angeles, CA 90048 USA; USC, Keck School of Medicine, Los Angeles, USA

## Abstract

Clinical use of adipose-derived stem cells (ASCs) for a variety of indications is rapidly expanding in medicine. Most commonly, ASCs are isolated at the point of care from lipoaspirate tissue as the stromal vascular fraction (SVF). The cells are immediately administered to the patient as an injection or used to enrich fat grafts. Isolation of ASCs from adipose tissue is a relatively simple process performed routinely in cell biology laboratories, but isolation at the point of care for immediate clinical administration requires special methodology to prevent contamination, ensure integrity of clinical research and comply with regulatory requirements. A lack of practical laboratory experience, regulatory uncertainty and a relative paucity of objective published data can make selection of the optimum separation method for specific indications a difficult task for the clinician and can discourage clinical adoption. In this paper, we discuss the processes which can be used to separate SVF cells from fat tissue. We compare the various mechanical and enzymatic methods. We discuss the practical considerations involved in selecting an appropriate method from a clinical perspective. Studies consistently show that breakdown of the extracellular matrix achieved with proteolytic enzymes affords significantly greater efficiency to the separation process. SVF isolated through mechanical methods is equally safe, less costly and less time consuming but the product contains a higher frequency of blood mononuclear cells and fewer progenitor cells. Mechanical methods can provide a low cost, rapid and simple alternative to enzymatic isolation methods, and are attractive when smaller quantities of ASCs are sufficient.

## Background

The clinical use of autologous adipose-derived stem cells (ASCs) is rapidly expanding because of promising results across a wide range of conditions. While progress in the use of cultured, modified and induced pluripotential cells has been measured in laboratory milestones, the use of autologous adipose-derived pluripotent cells is burgeoning at the clinical level. Clinical and pre-clinical studies show that autogenous ASCs demonstrably survive after transplantation, show pluripotential differentiation (Zuk et al. 2001; Planat-Benard et al. 2004; Naderi et al. 2014; Ude et al. 2014) and exhibit anti-apoptotic, anti-inflammatory, and angiogenic effects (Rehmam et al. 2004; Kapur and Katz 2013; Suga et al. 2010; Eto et al. 2012; Kato et al. 2014).

Applications as diverse as myocardial infarction, cosmetic surgery, osteoarthritis and bone regeneration, inflammatory bowel disease and chronic wounds are reported among a myriad of others (Savi et al. 2015; Matsumoto et al. 2006; Di Rocco et al. 2010; Asatrian et al. 2015; Nagaishi et al. 2015). There is some variation in the number of stem cells present in various donor sites and with donor age (Jurgens et al. 2008; Vilaboa et al. 2014; Buschmann et al. 2013). In general, the most efficient methods can isolate about 500,000–1,000,000 cells per gram of lipoaspirate tissue with a >80 % viability. The number of viable cells required for treatment of a particular condition is unknown because there is insufficient data to establish a reliable dose vs effect relationship. In general, because no additional adverse effects are reported with the use of autologous ASCs in fat grafting, the largest number of cells isolated at the point of care without expansion in culture is typically used. Despite a lack of reported clinical risk, in vitro studies have demonstrated potential oncological risks which clinicians should be cautious of when using SVF based therapies (Bertolini et al. 2012; Bielli et al. 2014).

The surge in clinical applications for ASCs increases the need for clear and reliable information about the efficiency, cost and safety of automated equipment and manual techniques which facilitate separation of the stromal vascular fraction (SVF) from adipose tissue. In clinical practice, adipose-derived stem cells are often not administered as a pure isolate but rather as one constituent of stromal vascular fraction, a heterogeneous mixture of cells resulting from the mechanical or enzymatic processing of aspirated adipose tissue. SVF contains a variety of cells including macrophages, various blood cells, pericytes, fibroblasts, smooth muscle cells, vascular endothelial progenitors and adipose-derived stem cells (Yoshimura et al. 2006; Bourin et al. 2013; Han et al. 2010; McIntosh et al. 2006; Bonab et al. 2006; Yoshimura et al. 2009). Stromal vascular fraction is one component of the heterogeneous mixture of adipose tissue fragments, stromal tissue, blood and tumescent fluid which constitutes lipoaspirate. The ASC content of SVF varies substantially depending on the method employed, with reports from less than 1 % of cells to over 15 % (Table 1). SVF cells can be safely isolated, quantified and characterized at the point of care in approximately 90 min. This is a timeframe which permits isolation and treatment to occur in the same surgical procedure, that is, at the point of care (POC).

**Table 1:** Summary of reported SVF isolation methods

References	Method summary	Mechanical or enzymatic	Automated, semi-automated, or manual	Total nucleated cells/cc lipoaspirate	ASC content	Viability
Baptista et al. (2009)	Lipoaspirate incubated with RBC lysis buffer for 15 min, then centrifuged 15 min at 900*g*	Mechanical	Manual	240,000	12,000/cc of lipoaspirate (5 %)	n/a
Shah et al. (2013)	Lipoaspirate vigorously shaken for 1–2 min with PBS. Infranatant saved. Repeated 2 times. Infranatant centrifuged 1200 rpm for 5 min	Mechanical	Manual	n/a	25,000/cc of lipoaspirate after culture	n/a
	Incubate adipose with 0.1 % collagenase at 37 °C for 1 h. Centrifuge 1200 rpm 10 min	Enzymatic	Manual	n/a	480,000/cc of lipoaspirate after culture	n/a
Markarian et al. (2014)	Lipoaspirate incubated with RBC lysis buffer for 15 min, then centrifuged 10 min at 600*g*	Mechanical	Manual	25,000	n/a	65 %
	Centrifuged lipoaspirate at 800*g* or 1280*g* for 15 min	Mechanical	Manual	10,000	n/a	70 %
	Lipoaspirate incubated with collagenase solution at 37C for 30 min. Centrifuge for 10 min at 600*g*	Enzymatic	Manual	350,000	n/a	65 %
Raposio et al. (2014)	Shake lipoaspirate in vibrating shaker for 6 min at 600 vpm. Centrifuge 6 min at 1600 rpm. Considered ASC to be any cell CD31^−^/CD34^+^/CD45^−^	Mechanical	Manual	125,000	6250/cc of lipoaspirate (5 %)	n/a
Mitchell et al. (2006)	Incubate lipoaspirate in 0.1 % collagenase for 60 min at 37 °C	Enzymatic	Manual	308,000	n/a	n/a
Aust et al. (2004)	Incubate lipoaspirate in 0.1 % collagenase for 45 min at 37 °C	Enzymatic	Manual	400,000	n/a	93.9 %
Yoshimura et al. (2006)	Incubate with 0.075 % Collagenase at 37 °C for 30 min with constant agitation	Enzymatic	Manual	1,310,000	n/a	n/a
Suga et al. (2010)	Incubate with 0.075 % Collagenase at 37 °C for 30 min with constant agitation	Enzymatic	Manual	100,000	n/a	n/a
Conde-Green et al. (2014)	High speed centrifugation or vortexing and centrifuging	Mechanical	Manual	11,500 –23,000	MSC frequency: 6–13 %	80–90 %
	Collagenase-based digestion	Enzymatic	Manual	230,000	MSC frequency: 60 %	80–90 %
Fraser et al. (2013)	Cytori Celution System	Enzymatic	Automated	360,000	1900 CFU-F/g (<1 %)	84.7 %
Lin et al. (2008)	Cytori Celution System	Enzymatic	Automated	295,000	CFU-F/g =1.6 %	86.6 %
Aronowitz et al. (2013)	Cytori Celution System	Enzymatic	Automated	240,000	39,000 CFU-F/g (16 %)	93 %
	PNC Multi-Station: 35U collagenase/50 mL lipoaspirate. Incubate 30 min at 37 °C with constant agitation. Centrifuge at 2000 rpm for 10 min	Enzymatic	Manual	107,000	6,000 CFU/g (5.6 %)	57 %
	CHA Biotech Cha-Station	Enzymatic	Semi-automated	5000	390 CFU-F/g (7.8 %)	87 %
	Medi-Kan Lipokit with MaxStem	Enzymatic	Semi-automated	35,000	615 CFU-F/g (1.7 %)	72 %
Doi et al. (2013)	Tissue Genesis Cell Isolation system	Enzymatic	Automated	702,000	n/a	80.7 %
	Lipoaspirate incubated with 0.075 % collagenase for 30 min at 37 °C with constant agitation, then centrifuged at 800*g* for 10 min	Enzymatic	Manual	701,000	n/a	82.4 %
Williams et al. (2013)	Tissue Genesis Cell Isolation System	Enzymatic	Automated	7,100,000	n/a	78 %
Güven et al. (2012)	Sepax Technology	Enzymatic	Automated	260,000	CFU-F frequency 14 %	>90 %
	Lipoaspirate incubated with 0.15 % (w/v) collagenase for 60 min at 37 °C with agitation	Enzymatic	Manual	160,000	CFU-F frequency 11 %	>90 %
Vilaboa et al. (2014)	GID SVF Platform	Enzymatic	Semi-automated	719,000	n/a	83 %
Millan et al. (2014)	StromaCell by Microaire	Mechanical	Semi-automated	140,000	n/a	87.3 %
	Lipoaspirate incubated in 0.2 % (w/v) collagenase for 90 min at 37 °C	Enzymatic	Manual	368,000	n/a	74.5 %
Wang et al. (2012)	Medi-Kan Lipokit	Enzymatic	Semi-automated	n/a	41.67 %	n/a

## Enzymatic methods

Enzymatic methods of isolating SVF cells from adipose tissue at the POC are based on a commonly used laboratory method of obtaining stem cells. The methods used to manually process adipose tissue using collagenase follow the same basic steps, but vary slightly in technique and reagents used. Lipoaspirate is washed 2–3 times using an aqueous salt solution such as PBS, Lactated Ringer’s solution, or Hank’s Balanced Salt Solution (HBSS). The washed lipoaspirate is then incubated with a collagenase solution of variable concentration and composition, depending on the method and tissue dissociation enzyme product used. Enzymatic digestion is typically carried out in a heated shaker to provide constant agitation at 37 °C for 30 min to 2 h. The digested adipose tissue is then centrifuged (speed/duration vary. See Table 1) which separates the processed lipoaspirate into three main layers, the oil/adipose tissue layer, the aqueous layer, and the pellet. The SVF is contained within the pellet, so the other layers are discarded, although SVF cells can be recovered from the aqueous layer (Yoshimura et al. 2006). The pellet is washed to remove any residual enzyme and filtered to remove tissue fragments and detritus. Collagenase-based enzymatic methods can be up to 1000 times more effective in SVF cell recovery than mechanical methods. Enzymatic methods are more efficient in isolating SVF cells because disruption of the collagen-based extracellular matrix (ECM) which binds together adipocytes and other cells of adipose tissue.

Tissue dissociation enzyme mixtures used for the separation process are usually a mixture of type I and type II collagenases isolated from *Clostridium histolyticum,* and various other proteolytic enzymes such as neutral protease (Dispase) (Fogarty and Griffin 1973; Griffin and Fogarty 1973) isolated from *P. polymyxa* or thermolysin (Ke et al. 2013) isolated from *G. stearothermophilus* or *B. thermoproteolyticus*, depending on the product used. Commonly enzymatic methods are carried out using tissue dissociation enzyme mixtures such as CIzyme™ AS (Vitacyte LLC, Indianapolis, Indiana) or Liberase™ Research Grade (Roche Diagnostics, Basel, Switzerland). CIzyme™ AS is a mixture of type I and type II clostridial collagenase and dispase. The Liberase™ Research Grade enzyme mixture recommended for adipose-tissue digestion is mixture of type I and type II clostridial collagenase and thermolysin. Mixtures of enzymes have been shown to yield more nucleated cells than using only one enzyme, a quality attributed to the synergistic effect of the proteolytic enzymes in the breakdown of the ECM (McCarthy et al. 2010, 2011; Breite et al. 2010); however collagenase is still frequently used as the sole proteolytic enzyme in methods using products such as Collagenase NB6 (SERVA Electrophoresis GmbH, Heidelberg, Germany) or Collagenase type I CLS 270 (Worthington Biochemical Corporation, Lakewood, NJ).

Published yields of viable, nucleated SVF cells achieved using manual, collagenase-based digestions range from 100,000 nucleated cells/cc to 1,300,000 nucleated cells/cc of lipoaspirate processed (Table 1). Equipment like the PNC Multi-Station (PNC International, Gyeonggi-do, Republic of Korea) is commercially available for use in the manual preparation of SVF. The PNC Multi-Station contains a centrifuge and heated shaker inside of a sterile biohood which allows the entire processing to be conducted in sterile conditions.

## Mechanical isolation methods

Mechanical methods for SVF isolation report significantly lower yields of nucleated cells/cc of lipoaspirate processed. Cell yields are reported from 10,000 nucleated cells/cc of lipoaspirate to 240,000 nucleated cells/cc of lipoaspirate (Table 1). Mechanical methods seek alternative non-enzymatic means of removing SVF cells from the adipose tissue and tend to be focused around washing and shaking/vibrating lipoaspirate followed by centrifugation in order to concentrate the SVF cells. All of the mechanical methods mentioned in this article contain a centrifugation step in order to concentrate the SVF cells. The composition of the cell populations recovered through simple centrifugation and other non-enzymatic methods have been shown to contain a greater frequency of peripheral blood mononuclear cells and a substantially lower number of progenitor cells (Conde-Green et al. 2014; Raposio et al. 2014; Shah et al. 2013). This is because ASCs are concentrated in the small and medium sized vascular structures of adipose tissue, and without enzymatic lysis of the collagen-based extracellular matrix many progenitor cells remain trapped within the vascular endothelium layers and connective tissue fragments in the lipoaspirate.

While enzymatic methods consistently yield higher cell counts with a higher frequency of progenitor cells, mechanical methods do offer some distinct advantages. The digestion of adipose tissue to disperse the cellular constituents prolongs the isolation time and can be fairly expensive, with costs of $2–$5 per gram of tissue processed using GMP grade enzymes (Aronowitz and Ellenhorn 2013). In settings where maximum numbers of progenitor cells are not critical, a non-enzymatic separation method like that of Raposio et al. can provide a cost-effective alternative (Raposio et al. 2014). Additionally, mechanical methods tend to offer a faster processing time, some less than 15 min, because they do not require the extra 30–120 min allotted for enzymatic digestion to occur.

## Mechanical vs enzymatic methods

In 2014, Raposio et al. reported a non-enzymatic method for SVF isolation (Raposio et al. 2014). This method involves shaking lipoaspirate in a vibrating shaker for 6 min at 600 vibrations per minute and then centrifuging at 1600 rpm for 6 min to isolate the SVF cells. Raposio et al. reported that they were able to isolate around 125,000 nucleated cells per cc of lipoaspirate processed, however only about 5 % of these cells were progenitor cells, with the other 95 % being predominantly blood cells and endothelial cells. In comparison, enzymatic methods have reported SVF yields with significantly higher numbers of progenitor cells, for example one automated collagenase-based isolation system which was shown to yield over 15 % progenitor cells in the SVF (Aronowitz and Ellenhorn 2013). The discrepancy in SVF composition was supported by the paper by Conde-Green et al. (2014). Conde-Green et al. compared a standard collagenase-based method to two different mechanical methods. They reported that both mechanical methods yielded SVF populations with lower nucleated cell counts and lower frequencies of progenitor cells than the manual, enzymatic approach examined.

In 2014, Markarian et al. compared a variety of processing methods for SVF isolation side by side, both enzymatic and mechanical. Collagenase-based digestion was shown to be the most effective in terms of cell recovery Markarian et al. (2014). They reported about 350,000 nucleated cells/cc of lipoaspirate processed using a collagenase-based method. Another method examined was a non-enzymatic method involving centrifugation of lipoaspirate at either 800 *g* or 1280 *g*. At both speeds tested, far fewer nucleated cells were isolated, with only about 10,000 nucleated cells recovered per cc of lipoaspirate. They report no significant difference in viability between the various methods they examined.

In 2009, Baptista et al. reported another manual, mechanical method (Baptista et al. 2009). In this method, lipoaspirate is incubated with red blood cell (RBC) lysis buffer (150 mM NH_4_Cl, 10 mM KHCO_3_, 1 mM EDTA) at 37 °C for 15 min and then centrifuged for 15 min at 900*g*. They reported an average yield of about 240,000 nucleated cells per cc of lipoaspirate processed, but only about 12,000 of these (5 %) were adipose-derived stem cells. This was supported by Shah et al. (2013). They compared a similar method using PBS instead of RBC lysis buffer with the common collagenase-based method. Shah et al. cultured samples from each method to determine ASC content. They reported that once samples reached 80–90 % confluence that an average of 25,000 adipose-derived stem cells per cc of lipoaspirate processed were found in the sample acquired using this mechanical method, but 480,000 adipose-derived stem cells per cc of lipoaspirate we found in the sample acquired using the enzymatic method. Additionally, Shah et al. observed that the cells acquired using collagenase proliferated much more quickly when cultured, requiring less than half the time to reach 80–90 % confluence (6 days vs 13 days). This method using RBC lysis buffer was also tested by Markarian et al. (2014). They however reported a much lower yield, only about 25,000 viable cells/cc lipoaspirate processed.

The differences resulting in the yields observed using mechanical and enzymatic methods can be partially attributed to the physical location of SVF cells in adipose tissue. The SVF cells, particularly the mesenchymal stem cells and pericytes, tend to be localized in the perivascular space (Baer and Geiger 2012). As demonstrated by Zimmerlin et al. in 2010, immunohistochemical and immunofluorescent analysis reveal a localization of ASC and pericytes in these perivascular niches (Zimmerlin et al. 2010). Mechanical methods of isolation do not afford the same release of cells from the perivascular spaces because the disruption of the extracellular matrix is significantly reduced compared to enzymatic methods, leaving many of the desired cells trapped in larger tissue fragments which are subsequently discarded. As a result, the composition of the SVF resulting from mechanical isolations tends to be deficient in CD34 expression. This relative CD34^+^ progenitor deficiency has been suggested as a contributing factor to longer culture times required to reach 80–90 % confluence, as demonstrated by Shah et al. (2013).

## Automated/semi-automated devices for SVF isolation

Due to increasing interest of SVF cells in the clinical setting, various fully automated and semi-automated devices for SVF cell isolation, both enzymatically and mechanically based, have been developed by companies hoping to capitalize on this relatively new cellular technology. These devices employ similar methods to manual enzymatic and mechanical methods, but under more controlled conditions. In efforts to improve the yield of SVF isolation, many companies have developed processing systems which seek to optimize the isolation process by reducing the human element and limiting loss of viability due to processing, while still adhering to the current Good Manufacturing Practices (cGMP) (FDA 2014a). Some of these devices have been able to isolate large numbers of cells, while other devices have been shown to be less impressive. These companies continue to improve the devices and technology so as to optimize the cellular recovery and viability. While many of the automated systems are currently too expensive for use in the lab setting, it is very possible that these automated systems could become a common item used to provide safe and effective cellular therapies to patients in the clinical setting. Many of these companies are actively pursuing clinical trials in order to clinically validate their devices and technologies while also providing cellular therapy to patients in need, like the upcoming STAR trial (Cytori Therapeutics 2015) for treatment of scleroderma by Cytori Therapeutics, Inc. which has received an IDE from the FDA and is currently enrolling patients as of August 2015.

These automated and semi-automated systems tend to be small self-contained systems which are able to carry out each step of the process with little or no interference from a technician. One of the main benefits offered by many of these systems is increased sterility through the use of a closed system. Once the lipoaspirate is added to the device, it remains in a sterile environment, unlike many manual methods. In some devices, such as the GID SVF platform (GID Europe, London, UK), the lipoaspirate is harvested directly into the system (Vilaboa et al. 2014). These devices are all slightly different, but ultimately seek to achieve the same goal.

The Cytori Celution system (Cytori Therapeutics, Inc., San Diego, CA) has been reported in multiple studies. The Celution system is a closed, fully automated system which employs Cytori’s proprietary enzyme blend, Celase. The Celution system is capable of processing up to 360 cc of lipoaspirate at one time. The Celution system has been consistently reported to yield between 240,000–360,000 nucleated cells/cc of lipoaspirate processed and 84–93 % viability, while also yielding a large population of progenitors (Table 1) (Aronowitz and Ellenhorn 2013; Lin et al. 2008; Fraser et al. 2013). The Celution system has been reported for use in a variety of clinical applications including treatment of lower extremity ulcers, treatment of cryptoglandular fistulae, and breast augmentation (Marino et al. 2013; Borowski et al. 2015; Kakamura and Ito 2011). The Celution system possesses the CE mark, but is not commercially available in the United States; however, Cytori does have a number of Investigational Drug Exemptions (IDE) for trials using its ADRC technology though. Cytori currently has five clinical trials underway for indications including scleroderma, knee osteoarthritis, urinary incontinence and cutaneous thermal injury.

Another device which has been described in literature is the GID SVF platform mentioned above. The GID SVF platform offers a completely disposable, single use, closed system process using its proprietary enzyme mixture, GIDzyme-2 (GID Europe 2015). The device can process up to 350 cc of dry adipose at one time. Vilaboa et al. (2014) reported that using the GID SVF platform they were able to isolate 719,000 nucleated cells/cc of lipoaspirate with 83 % viability. No information is provided pertaining to progenitor content or clinical applications. The GID SVF platform has received the CE mark for distribution in the European Economic Area (EEA).

A device also reporting high cellular yields is the Tissue Genesis Icellator Cell Isolation system (Tissue Genesis, Honolulu, HI). The Icellator system is an automated, closed system which uses the Tissue Genesis proprietary enzyme blend, Adipase (Tissue Genesis 2015). In 2013, Williams et al. reported a staggering 7.1 million viable SVF cells/mL of canine adipose tissue with 78 % viability processed using the Icellator system (Williams et al. 2013). Another study conducted by Doi et al. (2013) reported a lower, but still impressive yield of 702,000 nucleated cells/cc of lipoaspirate with 80.7 % viability. Doi et al. compared the Icellator system to a manual collagenase-based method using 0.075 % collagenase to digest adipose tissue. They reported that using this manual method they were able to isolate 701,000 nucleated cells/cc of lipoaspirate with 82.4 % viability. No information is provided pertaining to progenitor content. The Icellator system has not been evaluated by the FDA for use in humans.

The Sepax Technology from BioSafe America (Biosafe Group, Lake Geneva, Switzerland) is an enzymatic, fully-automated, closed system. While marketed primarily for cord blood, bone marrow, and peripheral blood processing (Biosafe America 2015), it has been reported for use with adipose tissue as well. Guven et al. (2012) reported a yield of 260,000 nucleated cells/cc of lipoaspirate processed with around 14 % CFU-F, which they compared to a manual, enzymatic method which was able to isolate 160,000 nucleated cells/cc of lipoaspirate with around 11 % CFU-F. Over 90 % viability was reported in both groups. The Sepax-2 system has received a CE mark, 510(k) approval from the FDA and approval from the SFDA in China for processing of cord blood, bone marrow, and peripheral blood, not adipose tissue.

The Lipokit (Medi-Kan Int., West Hollywood, CA) is another semi-automated, enzymatic system. The Lipokit is an all in one system for the harvest, processing and transplant of SVF which can be used with or without enzyme (LipoKit II infomation 2015). The Lipokit uses custom disposable centrifuge syringes for the processing and handling of lipoaspirate, primarily for fat grafting, but can be used for isolation of SVF cells as well. There are very few articles published using the Lipokit, and in these reports, results vary widely. A study by Wang et al. (2012), reported on the effects of using the Lipokit for cell-assisted lipotransfer procedures in 18 patients. They reported 41.67 % ASCs in the SVF, but no data on cell count or viability was able to be acquired from the article. This report was contradicted by Aronowitz et al. (2013), who reported a much lower ASC frequency (1.7 %) with a fairly low nucleated cell yield, only about 35,000 cells/cc of lipoaspirate processed. The Lipokit platform has a CE mark as well as 510 (k) approval from the FDA in the United States as a graft preparation system, but not as an isolation system for SVF cells.

There are fewer mechanical, automated and semi-automated devices available for SVF cell isolation because most mechanical isolations can be conducted using standard laboratory equipment, so there is less of a need for an all in one device. Multiple companies advertise automated and semi-automated, mechanical systems, but many do not have published articles to attest to the yields of these devices. In addition, many of those which have been developed have been deemed to be ineffective in the clinical setting, such as the Fastem/Corios system recently described by Domenis et al. (2015). Domenis compared three methods of SVF isolation and cell-enhanced fat graft preparation. Overall, they concluded that the two enzymatic methods examined, the Lipokit and the Celution system, resulted in significantly more nucleated cells and clonogenic and multipotent progenitor cells for fat graft enhancement, while the Fastem/Corios system was unable to isolate adequate cells to significantly enhance a fat graft. No numbers for nucleated cell count, viability, or progenitor cell content are clearly reported.

One mechanical, semi-automated device which has reported adequate yields is the StromaCell system (Microaire Aesthetics, Charlottesville, VA). The StromaCell system is a patented centrifuge canister which allows for lipoaspirate to be harvested directly into the canister and easy recovery of the SVF cells from the canister after centrifugation at 1000*g* for 10 min (MicroAire Aesthetics 2013). In a 2014 study by Millan et al. (2014), collagenase based digestion was compared to mechanical isolation using the StromaCell device for SVF isolation. While isolating fewer cells than the standard collagenase-based method (368,000 cells/cc of lipoaspirate vs 140,000 cells/cc of lipoaspirate), they did report similar compositions in terms of progenitor content when analyzed by flow cytometry.

The main drawback of many of these devices is the cost of operation. The closed, enzymatic systems can be very expensive, with some costing over $50,000 for the system. In addition to purchasing the device, many require single-use disposable kits which can cost hundreds or thousands of dollars for a single disposable kit in some cases. A mechanical system like the StromaCell offers the benefit of a closed sterile system and tends to be more affordable, but does not provide the superior yield afforded by the enzymatic systems such as the Cytori Celution system or the Tissue Genesis Icellator system. All of the systems mentioned here can be operated by a single trained technician at the point of care. The processing times vary between systems, with mechanical systems being in the 15–30 min range and the enzymatic systems ranging from about 60–90 min depending on the amount of tissue processed.

### Regulatory concerns

Many of the mechanical methods were initially developed in an attempt to isolate a population of cells which could be considered “minimally manipulated,” which many believed would allow them to circumvent a large amount of regulatory oversight by the United States Food and Drug Administration (FDA) and other regulatory agencies around the world. Enzymatic methods produce cell populations which the FDA considers to be “more than minimally manipulated,” causing them to be more heavily regulated as a drug, while the non-enzymatic methods were thought to be considered “minimally manipulated” due to the ambiguity of certain areas of previous regulatory documents. Recent non-binding draft guidances for industry from the FDA (2014b, c) which clarify the FDA’s stance on minimal manipulation and adipose tissue derived HCT/P’s seek to classify all methods of SVF isolation, both enzymatic and mechanical, as yielding “more than minimally manipulated” cells, and thereby classifying SVF as a drug.

## Conclusion

Methods used to isolate of pluripotential mesenchymal cells from adipose tissue at the point of care are of increasing importance in medicine as a large body of clinical research shows promise for a burgeoning number of conditions. Mechanical techniques, such as simple washing or centrifugation of lipoaspirate are effective in isolating ASCs. Mechanical methods are appealing because they are simple, quick and generally not associated with expensive equipment or disposables. While more expensive than mechanical options, enzymatic methods for the isolation of stromal vascular fraction cells from adipose tissue yield more nucleated cells with a higher number of progenitor cells per volume of lipoaspirate processed, but overall viability tends to be unaffected by processing method. While mechanical methods may be cost-effective in the laboratory setting, enzymatic methods provide a superior SVF output for use in the clinical setting. The method that a certain lab or facility uses ultimately depends upon their needs and financial capabilities. Labs and clinics with insufficient funding to use enzymatic methods or automated/semi-automated devices still have the option of pursuing mechanical methods. There are differences in the number of adipose stem cells present in the various adipose tissue deposits of an individual and significant variation between individuals but adipose tissue in general is a rich source of pluripotential mesenchymal cells.
